# PLGA‐PNIPAM Microspheres Loaded with the Gastrointestinal Nutrient NaB Ameliorate Cardiac Dysfunction by Activating Sirt3 in Acute Myocardial Infarction

**DOI:** 10.1002/advs.201600254

**Published:** 2016-10-24

**Authors:** Panke Cheng, Wen Zeng, Li Li, Da Huo, Lingqing Zeng, Ju Tan, Jingting Zhou, Jiansen Sun, Ge Liu, Yanzhao Li, Ge Guan, Yuxin Wang, Chuhong Zhu

**Affiliations:** ^1^ Department of AnatomyNational & Regional Engineering Laboratory of Tissue EngineeringState and Local Joint Engineering Laboratory For Vascular Implants, Key Lab for Biomechanics and Tissue Engineering of ChongqingThird Military Medical UniversityChongqing400038China

**Keywords:** fatty acids, ischemia, microspheres, Sirt3

## Abstract

Acute myocardial infarction (AMI) is the death of cardiomyocytes caused by a lack of energy due to ischemia. Nutrients supplied by the blood are the main source of cellular energy for cardiomyocytes. Sodium butyrate (NaB), a gastrointestinal nutrient, is a short‐chain fatty acid (butyric acid) that may act as an energy source in AMI therapy. Poly(lactic‐co‐glycolic acid)‐Poly (*N*‐isopropylacrylamide) microspheres loaded with NaB (PP‐N) are synthesized to prolong the release of NaB and are injected into ischemic zones in a Sprague–Dawley rat AMI model. Here, this study shows that PP‐N can significantly ameliorate cardiac dysfunction in AMI, and NaB can specially bind to Sirt3 structure, activating its deacetylation ability and inhibiting the generation of reactive oxygen species, autophagy, and angiogenesis promotion. The results indicate that NaB, acting as a nutrient, can protect cardiomyocytes in AMI. These results suggest that the gastrointestinal nutrient NaB may be a new therapy for AMI treatment, and PP‐N may be the ideal therapeutic regimen.

## 1 Introduction

The World Bank has estimated that the number of patients with myocardial infarction will increase to more than 23 million in China by 2030,^1^ and acute myocardial infarction (AMI) is the main cause of death.^2^ A lack of energy is the most significant cause of cardiomyocyte death in AMI, which then triggers other pathological changes. Once AMI occurs, the production of reactive oxygen species (ROS) at the infarction site quickly increases. These increase and then induce cardiomyocyte apoptosis and structural damage.^3^ The early stress and lack of energy caused by myocardial ischemia can trigger autophagy in AMI. However, this autophagy cannot last for a long time and is not sufficient to protect cardiomyocytes and endothelial cells. This limitation leads to cardiomyocyte death and vascular injury, which will further increase cardiac dysfunction.

The lack of energy caused by myocardial ischemia is the main reason for cell death in the infarcted region, which suggests that the timely replenishment of energy may be a better treatment for myocardial ischemia. As we know, when the body needs energy, the oral replenishment of nutrients is necessary and effective. Whether these gastrointestinally absorbed nutrients can also provide energy to the infarcted region in the treatment of AMI is unknown. Sodium butyrate (NaB, C_4_H_7_O_2_Na), is widely used in food and in the medical field. The active component of NaB is a short‐chain fatty acid (butyric acid), which is a gastrointestinal nutrient. Previous reports have demonstrated that NaB acts as an energy source for cellular differentiation and provides energy for the recovery of damaged cells.^4^, ^5^ NaB not only participates in the metabolism of fat and the production of ketone bodies but also in carbohydrate metabolism. This relationship means that NaB can act as an energy source and participate in cellular metabolism under aerobic or anaerobic conditions.^6^, ^7^

Because a persistent energy supply is essential for myocardial ischemia treatment, the release of nutrients at the damaged area is absolutely necessary. Poly(lactic‐co‐glycolic acid) (PLGA) is a copolymer,^8^ which possesses excellent biodegradability and biocompatibility properties because its ester linkages easily participate in hydrolysis, producing lactic acid, glycolic acid, and original monomers, which are nontoxic and easily metabolized through the krebs cycle in the body; moreover, the PLGA‐based microspheres have high stability, high structural integrity, tunable properties, and surface functionalization.^8^, ^9^ All of these characteristics allow its broad use in delivery systems. However, the limitations of PLGA‐based microspheres are its high burst release, poor drug loading, rapid degradation, and rapid drug release issues, as well as the small amount of time it remains in the body; these issues restrict its application, so that the hybrid PLGA delivery system is necessary. Poly (*N*‐isopropylacrylamide) (PNIPAM) is a temperature‐sensitive amphiphilic polymer that contains hydrophilic segments and hydrophobic segments.^10^, ^11^ When the temperature is lower than the critical solution temperature, the hydrogen‐bonding of hydrophilic segments controls the interaction between polymer chains and water, contributing to the polymer absorbing water and the subsequent dissolution of polymer chains and a swollen state formation; when the temperature is higher than the critical solution temperature, the hydrophobic interactions of the hydrophobic segments dominate the interaction between the polymer chains and water, and the polymer chains collapse, polymer precipitation occurs and a gel‐like state appears.^10^, ^11^, ^12^, ^13^ Based on the characteristics of PNIPAM, if the PNIPAM is enwrapped on the surface of PLGA microspheres, which can increase the encapsulation efficiency of PLGA by accelerating the microsphere solidification rate with the changing temperature, it can then also extend the drug release of PLGA by slowing its degradation.^14^ Moreover, the gel‐like characteristics of PNIPAM can contribute to the amount of time that PLGA microspheres remain in the body.^15^ This set of characteristics means that PLGA combined with PNIPAM may be an effective release material for drug release in the AMI treatment.

As the NaB acts as the energy supplier and participates in the cellular metabolism, the Sirt family (sirtuins) is involved in cell survival and metabolism. Sirt3, one subtype of the Sirt family, is located in the mitochondria, which indicates that NaB may be related to Sirt3. Previous reports have demonstrated that Sirt3 can promote the mobilization of cellular energy stores and protects cells in low‐energy states, acting as an important protector of cell viability.^16^ Whether Sirt3 plays an important protective role in AMI is unknown. Previous reports have demonstrated that Sirt1 activates Akt and targets endothelial nitric oxide synthase in acute states of induced cardiac angiogenesis^17^ and that Sirt3 destabilizes hypoxia‐inducible factor‐1α to reduce transcription of the proangiogenic gene vascular endothelial growth factor‐A (VEGF‐A) in cancer cell lines during hypoxia;^18^ however, there are no studies reporting the role of Sirt3 in cardiac angiogenesis.

In the present study, we demonstrated that PLGA‐PNIPAM microspheres loaded with NaB (PP‐N) microspheres could promote recovery after AMI by binding to the Sirt3 structure and promoting its deacetylation ability, followed by the inhibition of ROS generation and the initiation of autophagy as well angiogenesis, contributing to cardiomyocyte protection. As a result of these findings, we propose that the short‐chain fatty acid NaB, one type of gastrointestinal nutrient, may be a new agent for AMI treatment, and PP‐N may be the perfect drug delivery system.

## 2 Results and Discussion

**Figure 1:**
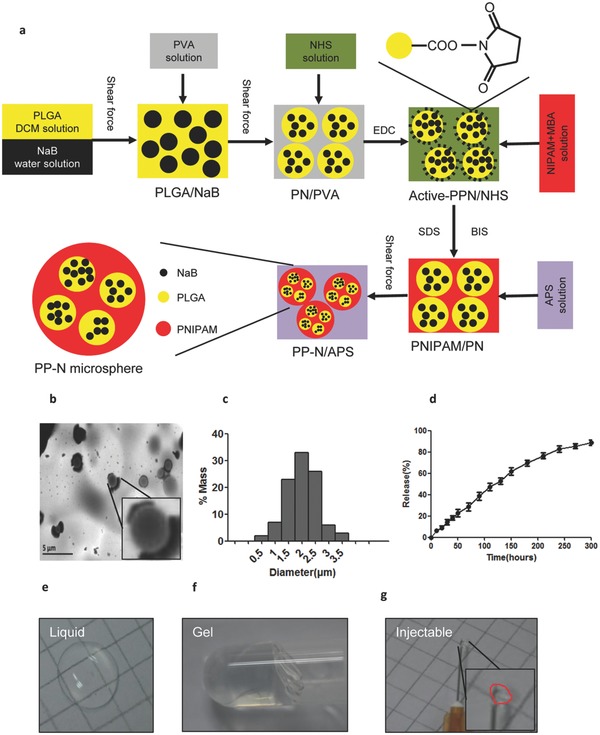
Characterization of PP‐N microspheres. a) PP‐N microspheres were synthetized as shown. NaB was enclosed in PLGA, forming the PLGA‐NaB microspheres, and the microspheres were enclosed in PNIPAM, forming the PP‐N microspheres. b) The size of the PP‐N microspheres. Representative TEM images of PP‐N microspheres. c) The size distribution of the particles. d) The release of NaB from the PP‐N microspheres. PP‐N microspheres were suspended in PBS at 37 °C at each measurement time point. PP‐N microsphere samples were measured using a UV–vis spectrometer with a wavelength of 428 nm. The PP‐N microspheres were the thermosensitive hydrogel, e) and when the temperature was lower than 30 °C, the PP‐N microspheres were in a liquid state. f) When the temperature was higher than 30 °C, it was in a gel‐like solid state, and it was liquid at lower temperatures, below 30 °C. g) Due to the thermosensitive properties of PP‐N microspheres, it was injectable through a 30 G needle.

Due to the need for long‐term therapy following AMI, the mode of drug delivery used was examined in this study, and PP‐N microspheres (PLGA‐PNIPAM microspheres loaded with NaB) were designed step by step as described in the schematic diagram (**Figure** **1**a). Figure 1b shows the size of the microspheres, and Figure 1c show the size distribution of the particles, which were nearly 2 μm for PP‐N microspheres, and the average porosity of the microspheres calculated to be (13.17 ± 1.15)%. Release experiments were performed to analyze the release ratio (Figure 1d), and the average loading efficiency of NaB was calculated to be (78.44 ± 7.15)%. To analyze the thermosensitive characteristics of the PP‐N microspheres, they were observed at different temperatures. PP‐N microspheres are in a liquid state (Figure 1e) when the temperature is below 30 °C, but they morph into a gel‐like solid state when the temperature is above 30 °C (Figure 1f). Because of the thermosensitive properties of PP‐N microspheres, they are injectable (Figure 1g). These results show that PLGA‐PNIPAM microspheres could act as the sustained release carrier of NaB, and the release time could extend to 10 d, which would ensure that NaB could be persistently released for a long time, contributing to a persistent NaB presence and avoiding multiple injections. The size of the PP‐N microspheres was (2.01 ± 0.12) μm, and the cell diameter was nearly 10–20 μm, which ensures that the microspheres could be absorb by cardiomyocyte and endothelial cells through endocytosis.^19^, ^20^, ^21^ Moreover, because the PP‐N microspheres are a thermosensitive hydrogel with a phase‐transition temperature of 30 °C and the animal's temperature is higher than 30 °C, the thermosensitive properties of PP‐N microspheres contribute to persistent NaB existence.

**Figure 2:**
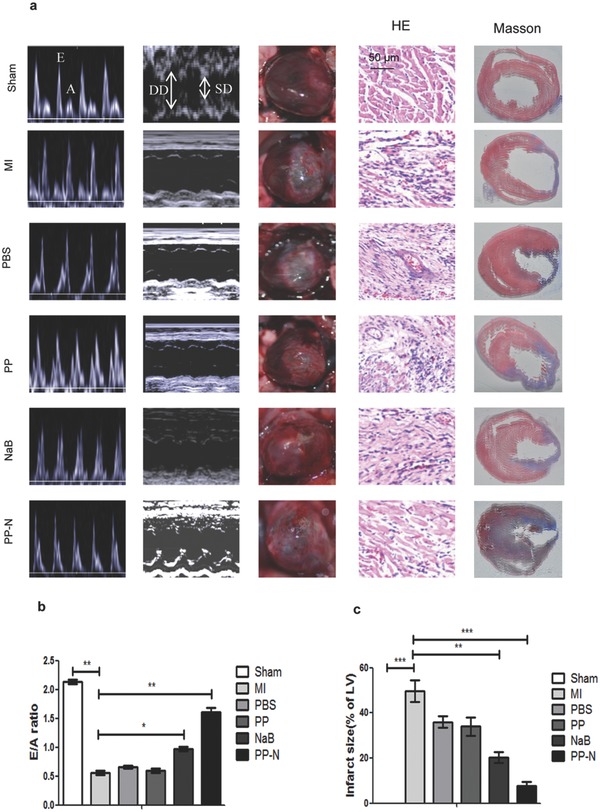
PP‐N ameliorates cardiac dysfunction. a) The echocardiogram, heart photography, hematoxylin‐eosin (HE) staining, and Masson staining of SD rats. SD rats were anesthetized with 30 mg kg^–1^ sodium pentobarbital at a concentration of 10 mg mL^–1^ through intraperitoneal injection. Pulsed‐wave Doppler echocardiography and M‐mode echocardiography of mitral inflow were performed. Four weeks later, the heart was photographed in situ, and the effects of different treatments were significant. HE and Masson staining of the heart sections. The blue staining represents the inflammatory cells, and the red staining represents the cardiomyocytes for HE staining. The blue staining represents the fibrotic tissue, and the red staining represents the cardiomyocytes for Masson staining. b) The statistical results of the E/A ratio. c) The statistical results of the degree of fibrosis shown by Masson staining. *P < 0.05; **P < 0.01; ***P < 0.005.

To detect the effect of PP‐N on AMI, a Sprague–Dawley (SD) rat AMI model was established and the myocardial infarction (MI) occurred, PBS, PP microspheres (PLGA‐PNIPAM microspheres) and PP‐N microspheres were injected into the ischemic zone of the heart through a multipoint injection. Four weeks later, echocardiography and cardiac catheterization were performed on the rats to analyze cardiac function. The systolic and diastolic function of the hearts were significantly improved in the rats that were injected with NaB and PP‐N microspheres, and the effect of PP‐N microsphere injection was greater than that of the NaB injection; the PP microsphere and PBS injection had no effect, and both NaB and PP‐N microspheres decreased the infiltration of inflammatory cells and the fibrosis ratio, as shown by pathological analysis and Masson staining, with the greatest effect from the PP‐N microspheres (**Figure** **2**a,c). Moreover, NaB and PP‐N microspheres reduced the left ventricular end‐diastolic dimension (LVEDD) and left ventricular end‐systolic dimension (LVESD) values and significantly increased the left ventricular ejection fraction (LVEF) % and left ventricular fractional shortening (LVFS)% and the ratio of peak early to late diastolic filling velocity (E/A ratio) in the rats (Figure 2a,b), and the effects of PP‐N microspheres were greater than those of NaB (**Table** **1**). NaB and PP‐N microspheres also increased the systolic blood pressure (SBP) and the diastolic blood pressure (DBP) in the rats and restored the LV systolic pressure (LVSP) and maximal rate of the increase of left ventricular pressure (+d*P*/d*t*), which were the LV systolic function indexes, to the basal levels. In addition, this treatment upregulated the LV end‐diastolic pressure (LVEDP) and the maximal rate of the decrease of left ventricular pressure (–d*P*/d*t*) values, which were the LV diastolic function indexes, and restored diastolic function. Again, the effect of the PP‐N microspheres was greater than that of NaB (Table 1). However, both NaB and PP‐N microspheres had no effect on the heart ratio (Table 1). These findings suggest that both NaB and PP‐N microspheres can improve cardiac function and inhibit fibrosis in AMI treatment, and the effect of PP‐N microspheres was greater than that of NaB alone because the PP‐N microspheres can slowly release NaB over a long time period. After AMI, the cell death and extracellular matrix degradation appearance led to the attenuation and collapse of the ventricular wall, which contribute to cardiac dysfunction. Although the NaB injection alone could save the dying cardiomyocytes and endothelial cells, it cannot reverse the cardiac extracellular matrix degradation and the changes of the ventricular wall. After the PP microspheres injection, because the body temperature was above 30 °C, PP microspheres could quickly become gel‐like and prop up the ventricular wall to avoid its collapse, but the PP microspheres had no additional function for cardiac function recovery because they did not load the drug. We thus designed the PP‐N microspheres for AMI treatment, as they could slowly release the NaB to achieve the effect of NaB and could also achieve the goal of propping up the ventricular wall, leading to improved cardiac function and inhibition of fibrosis.

**Table 1:** The indexes of cardiac function

Index	Sham	MI	PBS	PP	NaB	PP‐N
LVEDD [mm]	6.42 ± 0.35	9.92 ± 0.54a)	9.83 ± 0.44	9.43 ± 0.27	8.65 ± 0.49b)	7.26 ± 0.29b)
LVESD [mm]	3.1 ± 0.26	7.25 ± 0.35a)	7.12 ± 0.24	7.09 ± 0.48	5.8 ± 0.43b)	4.25 ± 0.51b)
LVEF [%]	72.9 ± 2.5	33.77 ± 3.15a)	33.63 ± 3.1	32.67 ± 2.88	40.5 ± 2.38b)	51.5 ± 2.06b)
LVFS [%]	57.74 ± 5.99	19.33 ± 4.09a)	22.45 ± 3.63	22.7 ± 4.48	33.4 ± 3.57b)	44.28 ± 3.62b)
HR [bmp]	510.33 ± 9.46	504 ± 10.8	512.67 ± 6.94	504.67 ± 6.34	506.33 ± 8.34b)	506.67 ± 8.26b)
SBP [mmHg]	168.67 ± 9.81	69 ± 7.26a)	72 ± 7.79	75.67 ± 5.31	97.33 ± 6.23b)	134 ± 8.64b)
DBP [mmHg]	98 ± 8.29	62 ± 4.55a)	62.67 ± 6.18	64 ± 5.72	80.33 ± 2.49b)	92.67 ± 4.78b)
LVEDP [mmHg]	6.53 ± 0.65	24.33 ± 3.21a)	22.47 ± 2.94	22.67 ± 4.47	17.47 ± 2.9b)	12.1 ± 1.26b)
LVSP [mmHg]	138.8 ± 5.99	49.3 ± 4.94a)	51.4 ± 6.75	55.2 ± 6.97	83.03 ± 4.93b)	119.2 ± 6.81b)
+d*p*/d*t* [mmHg s^–1^]	8734.67 ± 505.8	3779.33 ± 663.92a)	4391.33 ± 743.64	4137.67 ± 473.23	6254.67 ± 944.49b)	6946 ± 755.97b)
–d*p*/d*t* [mmHg s^–1^]	–9407.33 ± 663.81	–2969.7 ± 627.46a)	–3242.67 ± 576.69	–2850.33 ± 608.99	–4446 ± 483.27b)	–5893 ± 474.22b)

^a)^ Compared to the sham, *p* < 0.05

^b)^ Compared to the MI, *p* < 0.05.

**Figure 3:**
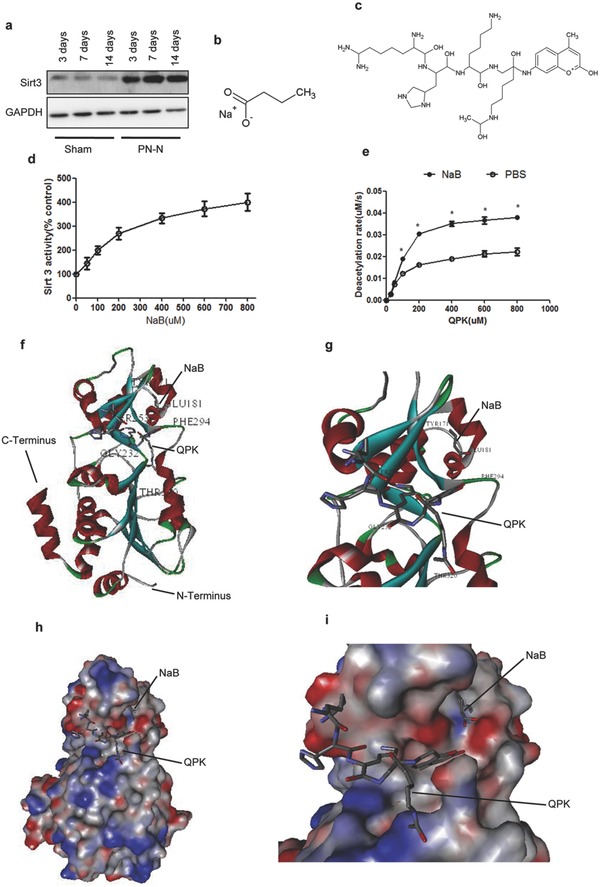
NaB bound to special structure in Sirt3. a) The cardiac tissue proteins from the ischemic marginal region collected at different days after PP‐N microspheres injection were assayed for Sirt3 by western blot. b) The molecules of NaB and c) QPK. d) The deacetylation activity of Sirt3 against QPK, which was detected with increasing NaB concentrations. e) The deacetylation rates of Sirt3 to QPK in the presence and absence of 200 × 10^–6^ m NaB. f) The overall structure of the Sirt3 complex bound with NaB and QPK. g) A closer view of the Sirt3 complex with the activator binding site, with the closer view showing that NaB and QPK have a direct interaction with each other. h) The surface representation of Sirt3 bound with NaB and QPK. i) A closer view of the surface representation of Sirt3, which was detailed for viewing clarity, and it significantly indicated that NaB and QPK were close to each other, which suggests that they could interact with each other. *compared to PBS, *P* < 0.05.

To clarify the mechanism of the PP‐N microspheres for AMI treatment, the energy metabolism‐related molecules of Sirt3 were analyzed. Interestingly, this study indicates that Sirt3 could be activated by NaB through binding to special structures in Sirt3. In the AMI, Sirt3 was activated after PP‐N microsphere injection, and the activation lasted for an extended time (**Figure** **3**a). To detect the effect of NaB (Figure 3b) on the activities of Sirt3, a peptide deacetylation assay was conducted. The P53‐derived substrate peptides QPKacytlK (QPK) with a C‐terminally attached fluorophore, which was the substrate of Sirt3, was selected for this assay (Figure 3c). The results show that NaB could significantly stimulate the Sirt3 deacetylase, which was dose dependent, and 200× 10^–6^ m may be the perfect concentration by being approximately 2.7‐fold against QPK (Figure 3d); the deacetylation rate of Sirt3 was significantly improved by NaB (Figure 3e). The molecular docking assay was conducted to rationalize the mechanism of the NaB‐activated Sirt3 in the QPK assay. In the Sirt3/QPK/NaB structure, the NaB bound close to the QPK and directly contacted the QPK fluorophore (Figure 3f,g), and the surface representation also indicated that NaB was bound near the binding site of QPK in Sirt3 (Figure 3h,i). The QPK fluorophore was packed with one surface formed by PHE294, its edge was closed to THR320, and its second plane would easily be a solvent with the absence of the activator. Importantly, NaB was bound between GLU181 and TYR171, which was bound near the binding site of QPK in Sirt3 and contracted the plane, which could inhibit the solution of the peptides (Figure 3f,g). The surface representation more visually displayed the positions of NaB and QPK, which were close to each other, and the structure suggested that the bonding site of NaB was near to the opening active site, trapping the bound QPK and increasing the interaction with the QPK peptide (Figure 3h,i). These results suggested that NaB could enhance the deacetylation rate of Sirt3 through special structural binding in Sirt3, and the bound sites were GLU181 and TYR171, which contributes to the interaction between Sirt3 and its substrate.

**Figure 4:**
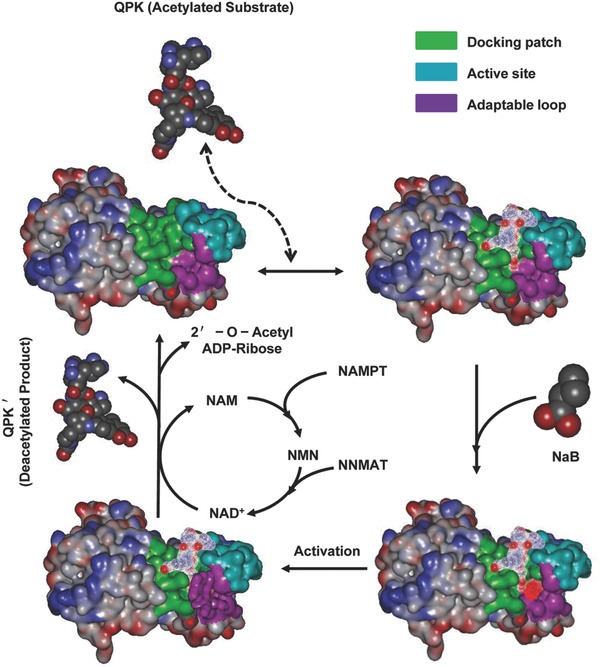
The model of deacetylated Sirt3 regulation by NaB. The substrate QPK binding to Sirt3 in the docking patch is unstable and reversible. Once NaB bound to Sirt3, which can induce the ordering of an adaptable loop, it contributes to the stabilization of the QPK/Sirt3 complex and promotes the QPK deacetylation in the active site of Sirt3. With the effect of NAD^+^, the loop was moved, the binding site was blocked, the NaB was released, and the deacetylated product (QPK′) was dissociated, releasing the 2′‐O‐ acetyl ADP‐ribose. Moreover, NAD^+^ was transferred into NAM and NMN and back to NAD^+^ with the enzyme‐catalyzed reaction of NAMPT and NMNAT.

To analyze the improvement of the deacetylation rate of Sirt3 through binding with NaB, the structure of Sirt3 and the deacetylation process of Sirt3 were analyzed. The structure of Sirt3 contains a “docking patch”, the substrate binding region in Sirt3, the “active site”, and the reaction zone of Sirt3, or the “adaptable loop”, which contribute to the binding and stabilization ability of the enzyme/substrate complex (**Figure** **4**). Due to the special structure of the substrate and the small molecule compound, although QPK binding to Sirt3 was reversible, after NaB was bound to Sirt3, the adaptable loop was ordered, leading to the stabilization of the Sirt3/QPK complex. The substrate was then properly deacetylated by the enzyme through stabilized exposure to the active site. In case of the hydrolyzing of NAD^+^ and transferring of the lysine‐bound acetyl group, the loop was moved, and the binding site was blocked, followed by activator release and deacetylated product dissociation. On the other hand, the hydrolyzed product of NAD^+^ was nicotinamide (NAM), and the transferred product of the lysine‐bound acetyl group was 2′‐O‐acetyl adenosine diphosphate(ADP)‐ribose; NAM was transferred to nicotinamide mononucleotide(NMN) by nicotinamide phosphoribosyl transferase (NAMPT), and then the NMN was transferred back to NAD^+^ by nicotinamide mononucleotide adenylyltransferase (NMNAT), forming the NAD^+^ cycle.^22^, ^23^ The above results indicated that NaB could contribute to the stabilization of the Sirt3/substrate complex by ordering the “adaptable loop”, leading to the deacetylation of the enzyme for the substrate through stabilized exposure to the “active site”. With the function of the NAD^+^ cycle, Sirt3 was reset, followed by activator release and deacetylated product dissociation, improving the deacetylation rate of Sirt3.

**Figure 5:**
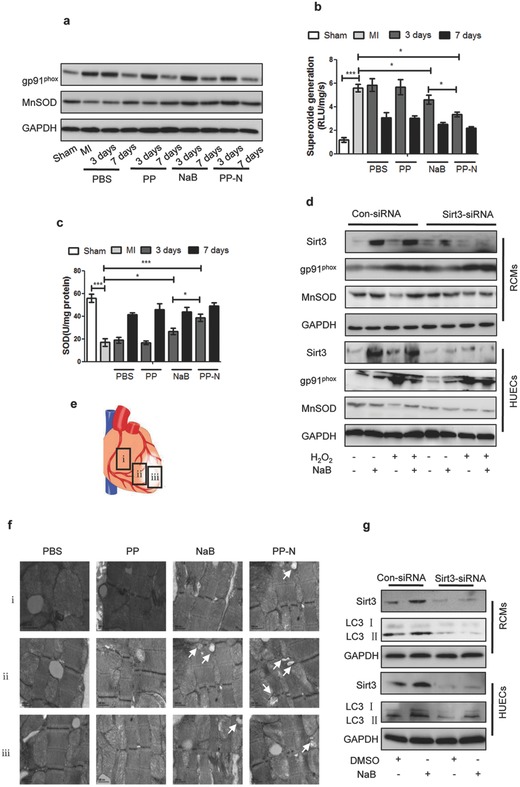
The effect of PP‐N on ROS generation and autophagy with Sirt3 activation. a) The cardiac tissue proteins from the ischemic marginal region collected at different days after injection were assayed by western blot. The statistical results of b) superoxide generation and c) SOD at different days after injection are shown. d) RCMs and HUVECs were infected with pSUPER vectors expressing Sirt3 siRNA or with control vectors as described in the protocol, the cells were pretreated with 150 × 10^–6^ m H_2_O_2_ for 4 h, and then 200 × 10^–6^ m NaB was added into the culture for another 24 h; after which, the cells were collected and analyzed by western blot assay. e) The heart tissue proteins were collected from the following different regions: (i) the nonischemic region, (ii) the ischemic marginal region, and (iii) the ischemic region. f) The heart tissues collected 3 d after injection were analyzed by TEM to detect autophagosomes. The arrows indicate autophagosomes. g) RCMs and HUVECs were infected with pSUPER vectors expressing Sirt3 siRNA or with control vectors as described in the protocol, and then 200 × 10^–6^ m NaB was added into the culture for 24 h; after which, the cells were collected and analyzed by western blot assay. *P < 0.05; ***P < 0.005.

**Figure 6:**
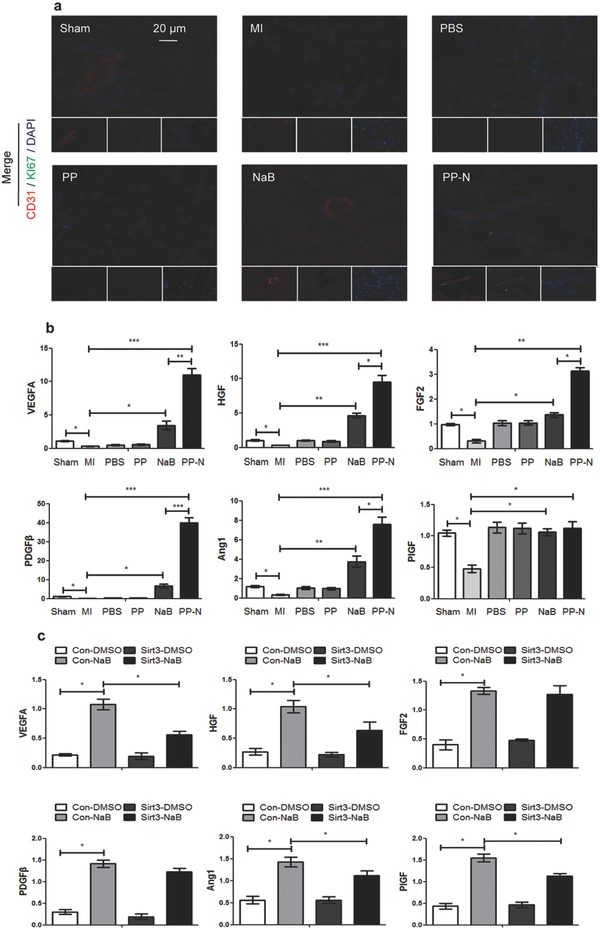
The effect of PP‐N on angiogenesis with Sirt3 activation. a) The heart sections collected at four weeks after injection were analyzed with immunofluorescence. They were stained for CD31 (red), a marker of endothelial cells, Ki67 (green), a marker of proliferation, and DAPI (blue), a maker of nuclei. b) qRT‐PCR analysis of the relative mRNA levels of angiogenesis‐related genes in the cardiac ischemic zone at four weeks after injection. c) RCMs were infected with pSUPER vectors expressing Sirt3 siRNA or with control vectors, as described in the protocol, and the cells were then treated with 200 × 10^–6^ m NaB for 24 h. The cytokines levels secreted by the RCMs were analyzed by enzyme‐linked immunosorbent assay (ELISA). *P < 0.05; **P < 0.01; ***P < 0.005.

In order to detect the biological function of PP‐N after NaB binding to Sirt3, ROS, autophagy, and angiogenesis were analyzed. The ROS marker NADPH oxidase subunits gp91phox and manganese superoxide dismutase (MnSOD) were significantly inhibited and promoted after PP‐N injection in AMI (**Figure** **5**a). Superoxide (Figure 5b) and superoxide dismutase (SOD) generation (Figure 5c) detection confirmed that PP‐N microspheres could significantly inhibit ROS generation through promoting antioxidant enzyme activity, and the in situ ROS production measurements and staining results of the redox‐sensitive compound dihydroethidium also confirmed the antioxidant ability of PP‐N (Figure S1, Supporting Information). Moreover, in vitro, for Sprague–Dawley rat cardiomyocytes (RCMs) that were isolated from 1‐day‐old neonatal SD rats and human umbilical cord vein endothelial cells (HUVECs), the Sirt3‐siRNA assay confirmed that Sirt3 was the key factor in PP‐N inhibited ROS generation (Figure 5d). The treated hearts were divided into 3 groups in the AMI model (Figure 5e). The transmission electron microscopy (TEM) assay indicated that PP‐N could significantly promote the number of autophagosomes (Figure 5f), and the western blot for LC3II protein levels and the immunofluorescence dot assay for LC3II‐GFP also reflected the PP‐N induced autophagy (Figures S2A and S2B, Supporting Information). In vitro, autophagy was induced by NaB, as shown by TEM (Figure S2C, Supporting Information) and western blot assay (Figure S2D, Supporting Information). The Sirt3‐siRNA assay confirms that Sirt3 was the key factor in PP‐N induced autophagy (Figure 5g and Figure S2E, Supporting Information). For apoptosis detection, PP‐N could significantly inhibit apoptosis according to the caspase3 protein assay, capase3 activity assay, and TUNEL assay in vivo (Figure S3, Supporting Information), and NaB also could significantly inhibit apoptosis according to the western blot assay for apoptosis markers detection, AnnexinV‐FITC, and propidium iodide assay, cell death and activity assay in vitro (Figure S4, Supporting Information). For the functional recovery of cardiac ischemic zones, the recovery of blood supply is the first need. To investigate the angiogenic ability of PP‐N, further experiments were performed. Immunofluorescence assays for the endothelial cell marker CD31 and the proliferative marker Ki67 were performed in cardiac ischemic zone four weeks after injection. The results show that PP‐N microspheres could stimulate endothelial cell proliferation (**Figure** **6**a) by promoting the mRNA expression of angiogenic factors (Figure 6b), such as VEGFA, hepatocyte growth factor, angiopoietin‐1, fibroblast growth factor‐2 (FGF2), and platelet derived growth factor beta (PDGFβ). In vitro, through EdU staining, the CCK8 kit and migration assays, the results show that NaB‐treated RCM culture supernatants (NaB‐RCM‐cus) could significantly promote endothelial cell proliferation, activity, and migration (Figure S5, Supporting Information). On the other hand, both NaB and NaB‐RCM‐cus had no effect on RCF proliferation, activity, and migration (Figure S6, Supporting Information). Moreover, when Sirt3 was knocked down in RCMs, the secretion of angiogenic factors other than PDGFβ and FGF2 were significantly inhibited in RCMs after NaB treatment (Figure 6c). These results suggested that the released NaB after PP‐N injection during AMI treatment could activate a series of biological functions through binding to Sirt3; the ROS generation was inhibited in infarct area, and the autophagy of cardiomyocytes and endothelial cells was enhanced. Moreover, in the infarct area, angiogenesis appeared with the secretion of an amount of angiogenic factors.

## 3 Conclusions

Currently, there are four types of microspheres used in the treatment of myocardial infarction, which contain polymers, liposome, superparamagnetism, and hydrogel. Biodegradable, hydrophobic polymers are the most common materials for microsphere design, and they contain a single material,^24^, ^25^, ^26^ such as PLGA, ketal, polylactic acid (PLA), and chitosan microspheres, or a composite material,^27^, ^28^, ^29^ such as PLGA‐PEI microspheres, the characteristics of these microspheres are biocompatibility, security, nontoxicity, and rapid degradation. The other microspheres are mainly loaded with polypeptides and siRNA,^30^, ^31^, ^32^ such as liposome microspheres, and the main features of these microspheres are rapid degradation and absorption by cells through endocytosis, which can contribute to the release of polypeptides and siRNA. Additionally, a category of microspheres acts as the scaffolds of the cell, carrying the functional cell to the infarct site for the myocardial infarction treatment,^33^, ^34^ such as superparamagnetic microspheres, and the characteristics of these microspheres are porosity and high adsorption capacity, resulting in stable conjugation with the target cell. In addition, a category of microspheres based on a temperature sensitive hydrogel are used in myocardial infarction treatment,^35^, ^36^, ^37^ such as PNIPAM and gelatin, and the characteristic of temperature sensitivity guarantees that these microspheres can avoid the problems of a difficult injection and nonlocation, moreover, the degradation rate of these microspheres is slow, which can contribute to the goal of long‐term and slow drug release. The PLGA‐PNIPAM hydrogel microspheres designed in the present study have the advantages of PLGA and PNIPAM, and which can avoid the disadvantages of low loading efficiency, rapid degradation, and easy movement. PLGA and PNIPAM are used to design the microspheres for NaB loading, forming PP‐N microspheres, which have good therapeutic effects for AMI treatment.

After AMI, PP‐N microspheres, which can release NaB for an extended period, were injected into cardiac ischemic zones and were found to ameliorate cardiac dysfunction. NaB bound to special structures in Sirt3 and promoted its deacetylation ability, triggering the biological function of Sirt3 and contributing to the protection of cardiomyocytes. Therefore, NaB was able to protect the damaged heart after AMI, and PP‐N microsphere delivery ensured that the effect of NaB persisted over an extended period. This study suggests that the nutrient NaB may be a new therapeutic agent following AMI and that other nutrients may also serve as therapeutic agents following AMI.

## 4 Experimental Section

*PP‐N and PP Microsphere Synthesis*: Solvent evaporation and a modified double‐emulsion technique were used to incorporate NaB into PLGA microspheres.^38^ Briefly, NaB (0.5 mg in 10 mL PLGA solution) was added to a PLGA solution (100 mg in 10 mL dichloromethane), and then this complex solution was emulsified in poly (vinyl alcohol) (2% w/v) to form the PLGA‐NaB microspheres. The PLGA‐NaB microspheres were incorporated with PNIPAM as previously reported.^39^ Briefly, using N‐(3‐dimethylaminopropyl)‐N‐ethylcarbodiimidehydrochloride and *N*‐hydroxysuccinimide (1:1) to activate the carboxyl functional groups of the PLGA‐NaB microspheres, the NIPAM, and *N*′‐methylene double acrylamide were copolymerized on the surface of these microspheres by sodium dodecyl sulfate and *N*,*N*′‐methylenebisacrylamide (BIS) and ammonium persulfate polymerization, forming the PP‐N microspheres. Similarly, PP microspheres that did not carry NaB were formed.

*Myocardial Infarction*: All SPF‐grade SD rats (180–230 g, *n* = 200) were purchased from the Experimental Animal Center of the Third Military Medical University. All the animal‐related procedures were approved by the Animal Care and Use Committee of the Third Military Medical University. SD rats were anesthetized with 50 mg kg^–1^ sodium pentobarbital at a concentration of 10 mg mL^–1^ administered through intraperitoneal injection. Using endotracheal intubation, thoracotomy was performed at the left fourth intercostal zone to occlude the left anterior descending coronary artery with a 6‐0 silk suture placed 2–3 mm distal to its origin. After 30 min, rats were injected with 100 μL of PBS that either lacked NaB (100 μL of the PP microsphere solution) or contained 5 μg NaB (100 μL of the PP‐N microsphere solution). A 30‐G needle was used to inject the solution into the ischemic region with a multipoint injection. Then, the chest was closed in layers, and the air in the chest was expelled. After the surgery, a series of experiments were preformed to evaluate the effect of the different treatments.

*Physiological Index Detection*: Physiological indexes were detected by echocardiography and cardiac catheterization. SD rats were anesthetized with 30 mg kg^–1^ sodium pentobarbital at a concentration of 10 mg mL^–1^ through intraperitoneal injection. Echocardiograms were recorded using a Vevo 770 imaging system (VisualSonics, Toronto, ON, Canada) equipped with a 30 MHz imaging transducer. Pulsed‐wave Doppler echocardiography and M‐mode echocardiography were used to detect mitral inflow as previously reported.^40^ After echocardiography, the left ventricular systolic and diastolic function parameters, peripheral circulation parameters, and cardiac pump function parameters were detected using a micromanometer‐tipped catheter (SPR 671, Millar Instruments, Houston, TX, USA) through the cannulation of the right carotid artery.

The complete detailed methods are provided in the online‐only Data Supplement.

## Supporting information

As a service to our authors and readers, this journal provides supporting information supplied by the authors. Such materials are peer reviewed and may be re‐organized for online delivery, but are not copy‐edited or typeset. Technical support issues arising from supporting information (other than missing files) should be addressed to the authors.

Supplementary
