# Supplementary material for: PLGA‐PNIPAM Microspheres Loaded with the Gastrointestinal Nutrient NaB Ameliorate Cardiac Dysfunction by Activating Sirt3 in Acute Myocardial Infarction
Source: Adv Sci (Weinh). 2016 Oct 24;3(12):1600254. doi: 10.1002/advs.201600254 (PMC5157182; doi:10.1002/advs.201600254)
Supplement: Supplementary file 1 — Supplementary [file ADVS-3-0-s001.pdf]

Copyright WILEY-VCH Verlag GmbH & Co. KGaA, 69469 Weinheim, Germany, 2016.

Supporting Information

for *Adv. Sci.,* DOI: 10.1002/advs.201600254

PLGA-PNIPAM Microspheres Loaded with the Gastrointestinal Nutrient NaB Ameliorate Cardiac Dysfunction by Activating Sirt3 in Acute Myocardial Infarction

*Panke Cheng, Wen Zeng, Li Li, Da Huo, Lingqing Zeng, Ju Tan, Jingting Zhou, Jiansen Sun, Ge Liu, Yanzhao Li, Ge Guan, Yuxin Wang, and Chuhong Zhu**

Supplement Data

PLGA-PNIPAM microspheres loaded with the gastrointestinal nutrient NaB ameliorate cardiac dysfunction by activating Sirt3 in acute myocardial infarction

Cheng. NaB ameliorates cardiac dysfunction

Panke Cheng, Wen Zeng, Li Li, Da Huo, Lingqing Zeng, Ju Tan, Jingting Zhou, Jiansen Sun, Ge Liu, Yanzhao Li, Ge Guan, Yuxin Wang, Chuhong Zhu †

Department of Anatomy, National& Regional Engineering Laboratory of Tissue Engineering, State and Local Joint Engineering Laboratory For Vascular Implants， Key Lab for Biomechanics and Tissue Engineering of Chongqing, Third Military Medical University, Chongqing 400038, China.

† Corresponding author Chuhong Zhu, Department of Anatomy, Third Military Medical University, Gao Tan Yan Street, Shaping Ba District, Chongqing, 400038, China.

E-mail: zhuch99@yahoo.com

Supplement Methods

NaB loading and release ratio

During the emulsion process, 0.5 mg NaB was loaded into the PP-N microspheres, and the loading ratio was evaluated using the following formula: Loading ratio=(the total NaB amount – the supernatant NaB amount)/(the total NaB amount)×100. The NaB release studies were conducted using the following steps: 1 ml of the PN-N microsphere solution (contained 0.05 mg NaB) was suspended in PBS at 37°C to analyze the release ratio of NaB from the PN-N microspheres at each time point. The NaB release sample was collected and analyzed using a UV-Vis spectrometer at a wave length of 428 nm.

Material characterization detection

The size distribution of the microspheres were measured by Accusizer 780/APS(Particle Sizing System, Santa Barbara, USA), microspheres were dispersed in the 0.2% poly (vinyl alcohol) solution and the size was measured. The porosity of the PP-N microspheres were detected by Mercury Porosimeter (AutoPore IV 9500, Micromeritics). 100mg PP-N microspheres were added into the porosimeter and tested with the pressure of 0.45 psi, and the details were recorded. Porosity = bulk density/apparent (skeletal) density × 100.

Tissue collection, immunofluorescence and staining

Rats were sacrificed after MI or sham operations at different time points and the hearts were obtained. After protein isolation following a standard protocol, heart tissues were fixed in 4% paraformaldehyde and embedded in paraffin. Tissues were cut into 5-μm-thick sections and then stained with Masson’s trichrome(Sigma, Gillingham, UK) or hematoxylin-eosin(HE) so that regions with fibrosis would appear blue. Sections were permeabilized with 0.5% Triton X-100, blocked with rabbit serum, and incubated with LC3,Troponin-I,CD31 and KI67(Santa Cruz, CA,USA).Fluorescence was detected by confocal laser scanning microscopy (Zeiss, Oberkochen, Germany). Portions heart tissue were also immediately embedded in optimum cutting temperature compound(Tissue-Tek) and stored at -80°C. These unfixed, frozen heart tissues were cut into 5-μm-thick sections.DHE staining(Sigma, Gillingham, UK) and TUNEL staining(Millipore, Billerica,MA,USA) were

conducted and analyzed with confocal laser scanning microscopy (Zeiss, Oberkochen, Germany).

Superoxide production, SOD and caspase-3 activity detection

The level of oxidative stress can be reflected by the superoxide production index, and tissue superoxide production was detected by lucigenin-enhanced chemiluminescence using the protocol previously described.[[1],[1]] The results were quantified as the relative light units per milligram protein per second. SOD activity was detected using the Superoxide Dismutase (SOD) Activity Assay Kit (Biovision, CA, USA) following the manufacturer'sprotocol,and the results were quantified as the relative light units per milligram protein. Caspase-3 activity was detected using a caspase-3activity assay kit (Millipore, Billerica,MA,USA) following the manufacturer's protocol, and the results were quantified as the nmol pNA per hour per milligram protein.

Cell culture

Rat cardiomyocytes (RCMs) and cardiac fibroblast (RCFs) were isolated from 1-day-old neonatal SD rat according to the previously reported method.[[2]] Human umbilical cord vein endothelial cells (HUECs) were purchased from American Type Culture Collection (ATCC). RCMs were cultured in DMEM medium, RCFs were cultured in F12 medium and HUECs were cultured in DMEM/F12 medium. All culturing media contained 10% fetal bovine serum(FBS),2 Mm L-glutamine, 100U/ml penicillin and 100 mg/ml streptomycin. Cells were cultured at 37°C with 5% CO_2.The cell-culture regents were purchased from Gibco (Grand Island, NE, USA).

Western blot and immunoprecipitation

Tissues and cells were lysed using RIPA lysing buffer (Beyotime,Beijing, China), and protein concentration was measured using a BCA protein assay kit (Beyotime,Beijing, China). Proteins were separated using SDS-PAGE and blotted onto PVDF membranes, which were incubated with primary antibodies for 12 h at 4°C and then with secondary antibodies for 1 h at 37°C.Bands were imaged using the FluorChem E data system(ProteinSimple, CA, USA). Each assay was repeated at least three times. For immunoprecipitation, the indicated antibody was coupled with protein G-Sepharose (Sigma, Gillingham,

UK) in 1% BSA in PBST, and then the antibody-conjugated beads were added into the cell lysates and incubated at 4°C for 2 h. After incubation, the beads were washed with PBS 3 times for further analyses.

Quantitative real-time PCR

Total RNA was isolated from tissues or cells by TRIzol reagent (Invitrogen, CA, USA), and the cDNA were synthesized using a QuantiTect reverse transcription kit (Takara Biotechnology, Tokyo, Japan).Real-time PCR was performed with KAPA SYBR Fast qPCR Kit(KAPA Biosystems, MA, USA), and the data were normalized relative to GAPDH. The primer sequences were purchased from Invitrogen (Invitrogen, CA, USA), and the relative differences between the assayed groups were calculated with REST MCS software. All the primer sequences were as the supplement table.

Transmission electron microscopy (TEM)

Heart tissues or PP-N microspheres were processed as previously described for TEM assay.[[3]] TEM was performed using an H-7000FA TEM an 80-kV accelerating voltage (Hitachi, Tokyo, Japan). Images were acquired with a digital camera attached to the TEM.

Vector transfection

Lentiviral vectors carrying GFP, LC3 cDNA and pSUPER vectors expressing either Sirt3 siRNA or Vps34 siRNA or with control vectors were constructed by GeneChem (GeneChem, Shanghai, China). Cells were transfected with the vectors using Lipofectamine 2000 (Invitrogen Life Technologies, CA, USA) following the manufacturer’s protocol.

Cell vitality, proliferation, death and apoptosis assay

Cell vitality was detected using the Cell Counting Kit-8(Beyotime,Beijing, China) and the Cell-LightTM EdU DNA Cell Proliferation Kit was performed to cell proliferation detection(RiboBio,Guangzhou, China) following the manufacturer’s protocols. Cell death ratio was measured by trypan blue assay. AnnexinV-FITC and propidiumiodide (PI) staining (Beyotime,Beijing, China) were used to detect apoptosis, and the apoptosis ratio was analyzed using a FACScan flow cytometer.

Cell migration assay

Transwell chambers (8μm, 24-well plate) (Corning, NY, USA) were performed to measure cell migration. For the Transwell chambers, membranes inserted in the lower chamber were coated with diluted Matrigel(BD Biosciences, NJ, USA), and cells were incubated in the upper chamber for 24 h, the lower chamber was added into different drug or RCMs culture medium. The insert membranes were cut out and cells were stained with crystal violet, counted, and photographed to determine the number of invading cells.

ELISA assay

RCMs were treated in different ways and for specific times, and the culture medium was collected for ELISA according to the manufacturer's instructions to detect vascular growth factors.

The cloning, expression and purification of Sirt3

The cloning, expression and purification of Sirt3 was done as described previously.[4] The catalytic cores of Sirt3(118-399) was PCR amplified, and then clone into pVFT3s, harvesting a construct with His+thioredoxin tag. BL21-CodonPlus (DE3) cells were used to proteins expression in LB medium through adding 0.5 mM isopropyl-β-D-thiogalactopyranosid at OD600 0.6. And then incubating over night at 15□, cells were disrupted by ultrasonic cell crusher, and centrifugation to remove the cell debris, the harvested the supernatant and added 10mM imidazole, incubation with talon resin at 4□ for other 1h. Washing the resin with 500mM NaCl, 50mM Tris/HCl, PH 7.8 (20 volumes) and 200mM NaCl, 50mM Tris/HCl, PH 7.8, 5mM imidazol (20 volumes). Recombinant proteins were eluted by 200mM NaCl, 50mM Tris/HCl, PH 7.8 ,250mM imidazol , and subjected to gel filtration with Superose 12 column (GE Healthcare, Waukesha, USA) in 150mM NaCl, 20mM Tris/HCl, PH 7.8, concentrated and stored at -80□. For activity assay of Sirt3, the protein was harvested by His tag as previous report.[6]

Sirt3 activity assay

Deacylase activity of Sirt3 was detected by commercial fluorescence assay kits (Biomol, PlymouthMeeting, USA) which

containing p53 derived substrate peptides QPKacetylK(QPK, the Sirt3 substrate), and the C-terminally attached fluorophore. 0.5ug Sirt3, 0.1mM QPK and 0.5mM were incubated at 37℃ as the protocol described. And followed the protocol, detection the reaction products. Depending on the the linear portion of the curves, calculating the velocity, and obtaining the deacetylation rate.

Statistical Analysis

The data are expressed as the mean±s.d. Fisher´s test was used for the statistical analyses. The nonparametric Mann-Whitney rank-sum test was used to determine differences between samples. One-way ANOVA followed by Bonferroni post hoc testing were also used for specific experiment analyses. A threshold of $P<0.05$ was used to determine statistical significance.

Supplement References

[1] Lund DD, Faraci FM, Miller FJ Jr, Heistad DD. Circulation **2000**, 101,1027.

[2] Cho HJ, Lee N, Lee JY, Choi YJ, Ii M, Wecker A, Jeong JO, Curry C, Qin G, Yoon YS. The Journal of experimental medicine **2007**, 204,3257.

[3] Wang L, Feng ZP, Kondo CS, Sheldon RS, Duff HJ. Circ Res **1996**, 79,79.

[4] Mikhaylova O, Stratton Y, Hall D, Kellner E, Ehmer B, Drew AF, Gallo CA, Plas DR, Biesiada J, Meller J, Czyzyk-Krzeska MF. Cancer Cell **2012**, 21,532.

[5] Gertz M, Nguyen GT, Fischer F, Suenkel B, Schlicker C, Fränzel B, Tomaschewski J, Aladini F, Becker C, Wolters D, Steegborn C. PLoS One **2012**,7,e49761.

[6] Schlicker C, Gertz M, Papatheodorou P, Kachholz B, Becker CF, Steegborn C. J Mol Biol **2008**,382,790.

Supplement Table

Oligonucleotide primers used for mRNA expression analysis by real-time RT PCR.

VEGFA	Forward	CTGGAGCGTATGTGACAAGC
	Reverse	GTCGGGATCCAAGTGAGGAC
HGF	Forward	ACAGCTTTTTGCCTTCGAGC
	Reverse	GCAAGAATTTGTGCCGGTGT
Ang1	Forward	CACGCTGAACGGTTACACAG
	Reverse	GTTTGACGCTCTCCCCGTTA
FGF2	Forward	CAACACTTACCGGTCACGGA
	Reverse	CCCCGTTTTGGATCCGAGTT
PGF	Forward	GAGGAACCCCACCTGTGATG
	Reverse	ATTCAGCAGGGACGAGTTGG
PDGFβ	Forward	CCTTTTAGCCCCACGAGTCC
	Reverse	CTTCTGTTGCTCCAGCAGGGA
GAPDH	Forward	GGTGGACCTCATGGCCTACA
	Reverse	CTCTCTTGCTCTCAGTATCCTTGCT

Supplement Figure Legends

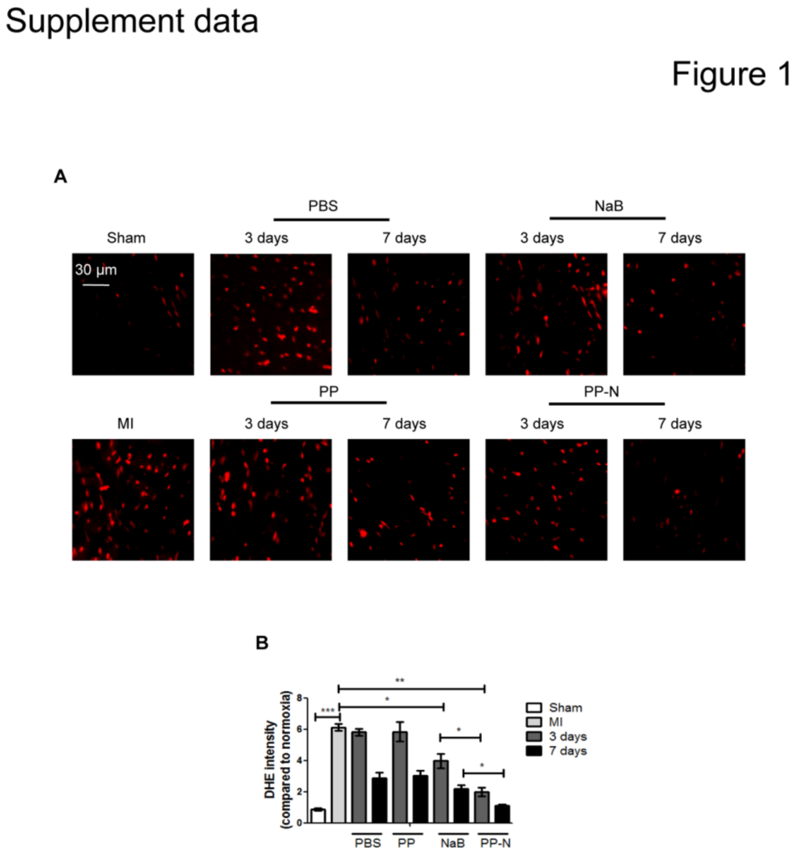

Figure S1. PP-N inhibits ROS generation in the cardiac ischemic marginal region. (A) The DHE intensity was measured and statistic through DHE staining analysis at different days after injection, **(b)**and obtaining the statistical data. *P<0.05;**P<0.01; ***P<0.005

Supplement data

Figure 2

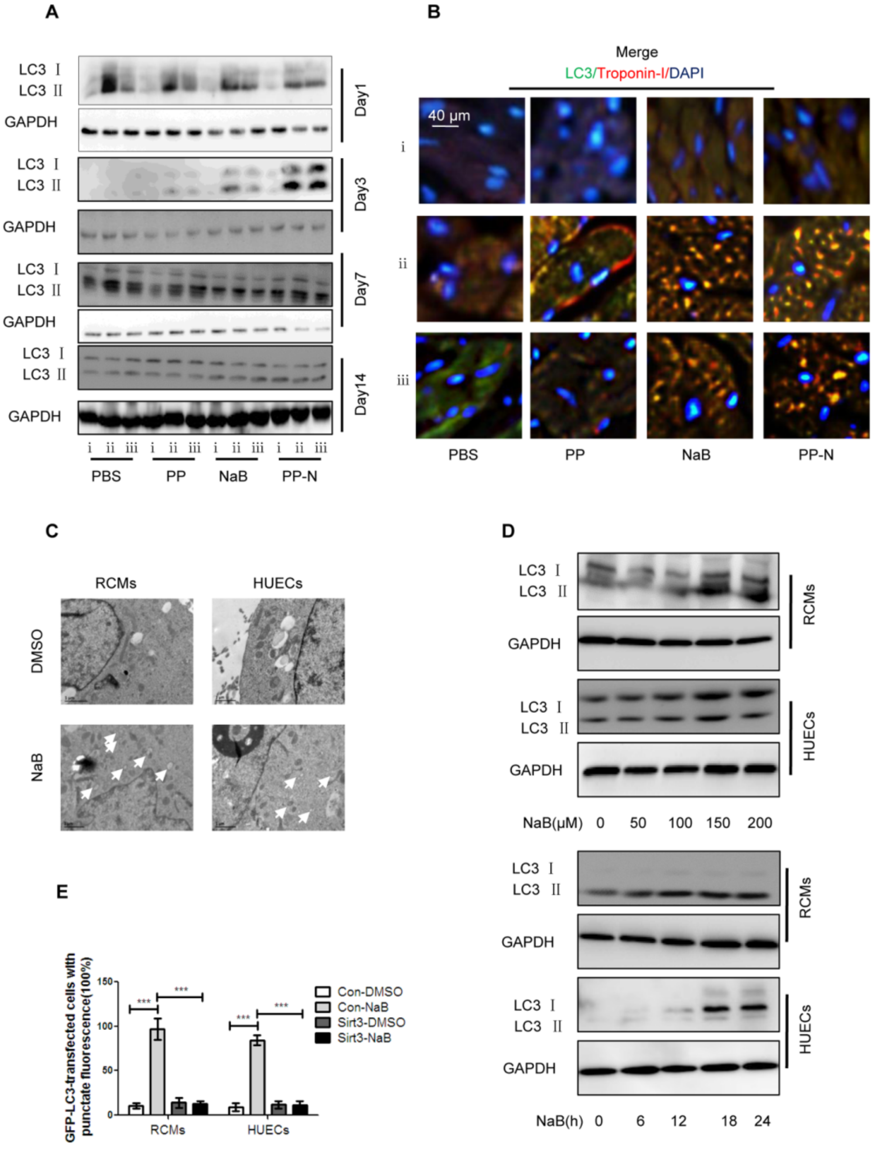

Figure S2. PP-N induces autophagy in cardiac ischemic regions through Sirt3 activation in vivo and vitro. (A) The heart tissue proteins collected at different days after injection were assayed with western blot analysis. **(B)** The heart sections collected at 3 days after injection were analyzed with immunofluorescence. Staining was conducted for LC3 (green), the marker of autophagy, troponin-I (red), the marker of cardiomyocytes, and DAPI(blue), the marker of nuclei. **(C)** RCMs and HUECs were treated by 200μM NaB 24 h, and then TEM was conducted to detect autophagosomes, the arrows indicate autophagosomes. **(D)** RCMs and HUECs were cultured by different dose NaB for 24h or 200μM NaB for different time,

LC3 was detected by western blot, and the NaB-induced autophagy was dose- and time-depend. **(E)** RCMs and HUECs were infected with GFP-LC3 vectors, with control vectors or pSUPER vectors expressing Sirt3, and the cells were then treated with 200μM NaB for 24 h. The GFP-LC3 dots were counted. ***P<0.005

Supplement data

Figure 3

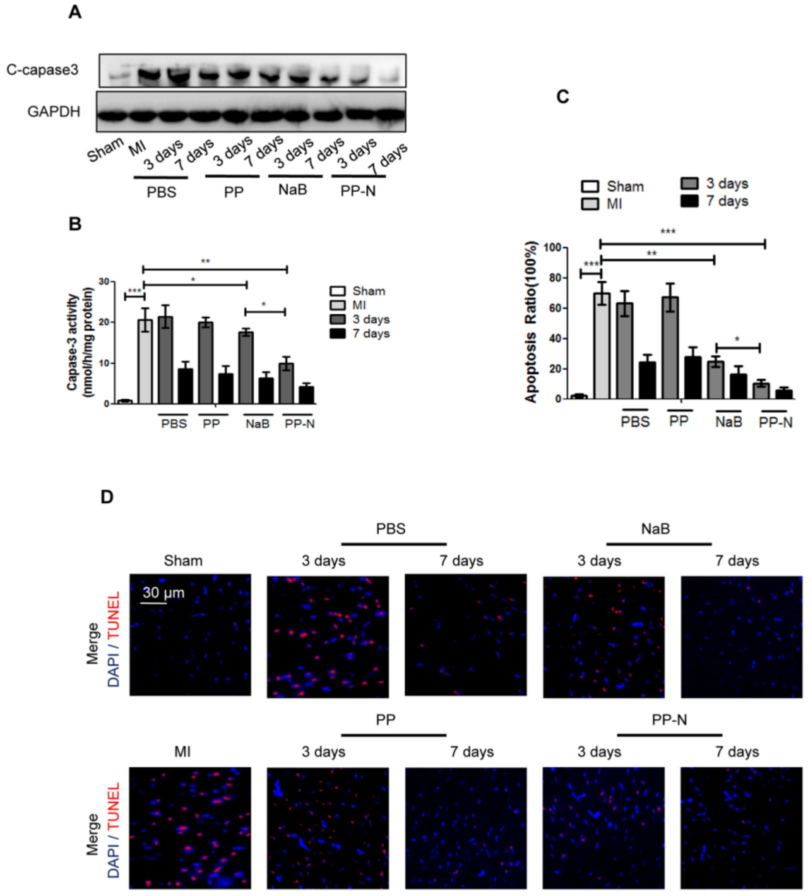

Figure S3. PP-N inhibits apoptosis in the cardiac ischemic marginal region. (A) C-capase3 was detected through western-blot assay with the cardiac tissue proteins from the ischemic marginal region collected at different days after injection. **(B)**The statistical results of caspase-3 activity at different days after injection was measured. **(C)** Apoptotic cells were counted through **(D)**TUNEL staining(in suit) of heart sections at different days after injection, TUNEL(red) and

DAPI(blue) stains were used. *P<0.05;**P<0.01; ***P<0.005

Supplement data

Figure 4

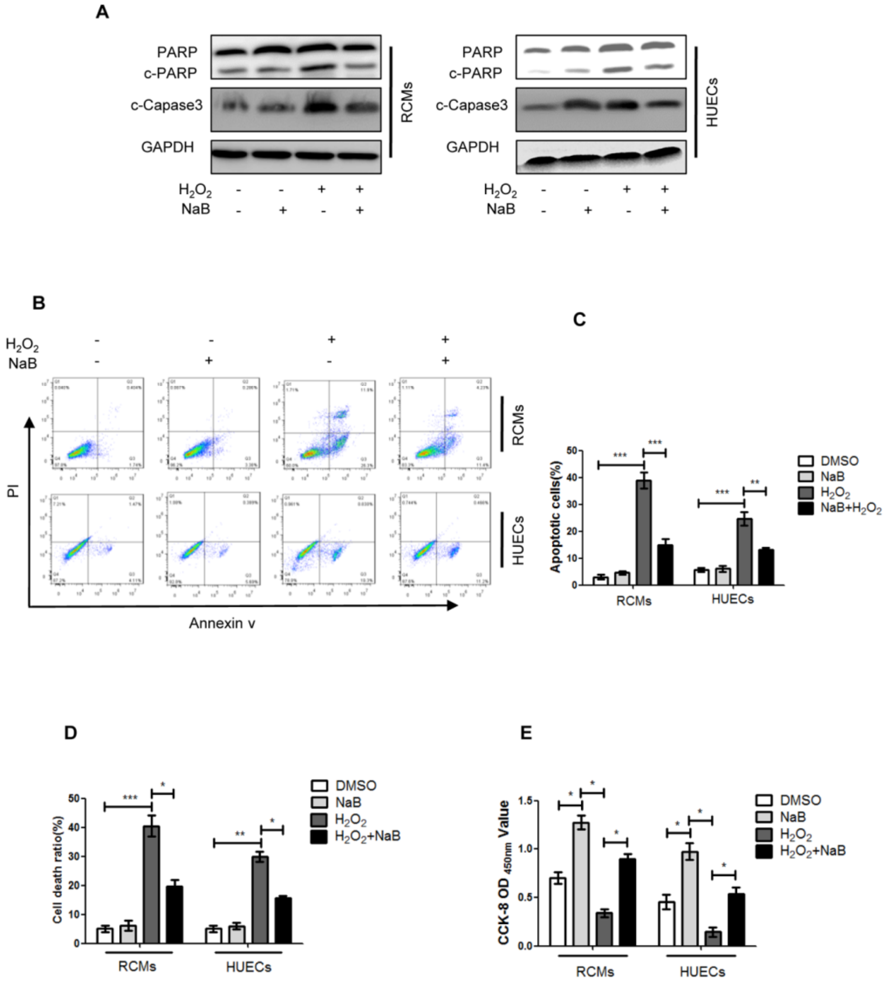

Figure S4. NaB inhibits apoptosis and activity in RCMs and HUECs. RCMs and HUECs were pretreated with150μM H_2O_2 for 4 h and then 200μM NaB was added into the culture for another 24 h. Cells were collected for **(A)** western blot assay or **(B)** AnnexinV-FITC and PI assay. **(C)** Thestatistical results of AnnexinV-FITC and PI. Cell death ratio and vitality were conducted by **(D)** trypan blue assay and **(E)** Cell Counting Kit-8, the total death rate=(the number of dead cell/total cell) ×100%. *P<0.05;**P<0.01; ***P<0.005

Supplement data

Figure 5

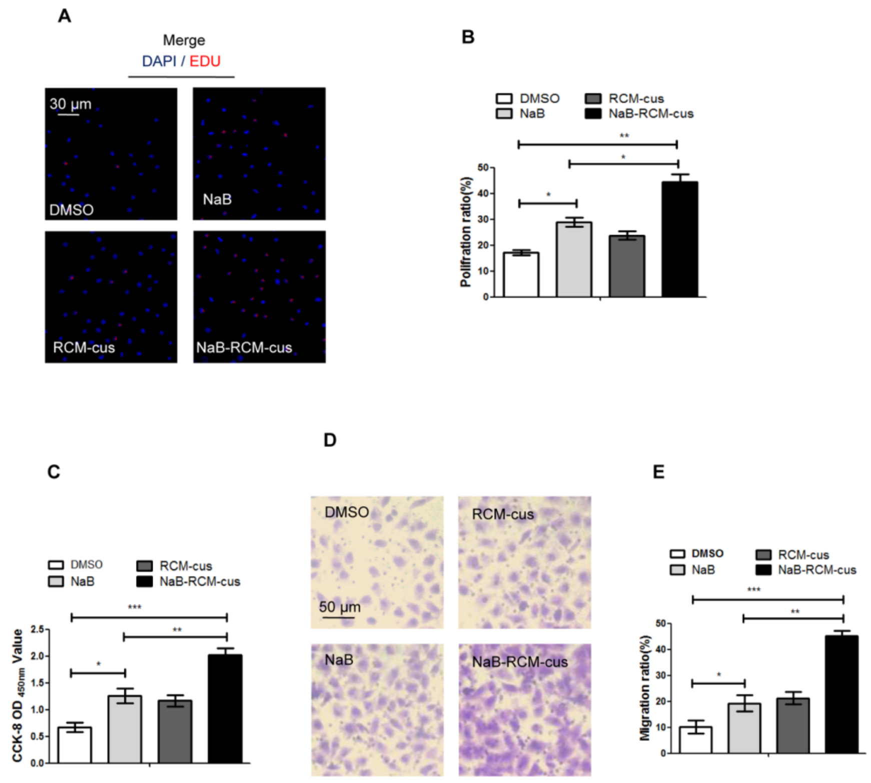

Figure S5. NaB treated RCM culture supernatants promote the proliferation, activity and migration of HUECs. HUECs were cultured with 200μM NaB, RCM culture supernatants (RCM-cus) or 200μM NaB treated RCM-cus (NaB-RCM-cus), and the cells were analyzed with **(A,B)** EDU staining, **(C)** CCK8assays and **(D,E)** Transwell. The statistical results of **(B)** EDU staining and **(E)** Transwell assays were obtained. *P<0.05;**P<0.01; ***P<0.005

Supplement data

Figure 6

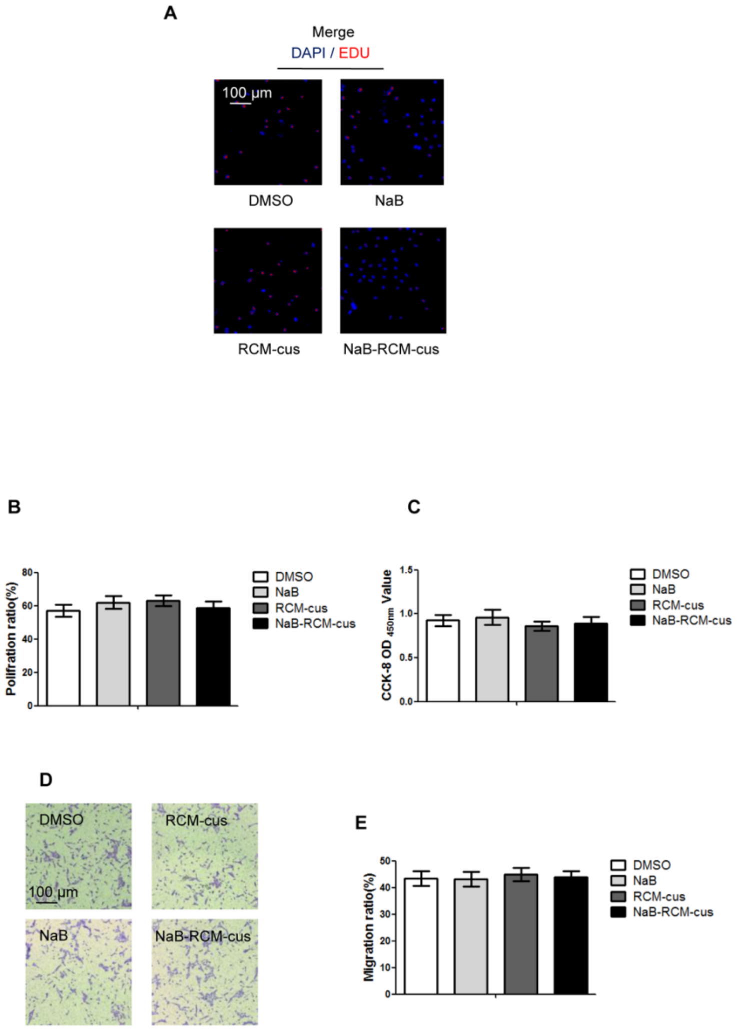

Figure S6. RCM paracrine has no effect on vitality, proliferation and migration of RCFs. RCFs were cultured by different RCM culture supernatant, **(A) (B)** cell proliferation, **(C)** vitality, **(D) (E)** migration and invasion was measured by Cell-LightTM EdU DNA Cell Proliferation Kit, Cell Counting Kit-8 and Transwell chambers. However, these indexes have no difference with different RCM culture supernatant treatment.
